# Which Medication Is the Patient Taking at Admission to the Emergency Ward? Still Unclear Despite the Swedish Prescribed Drug Register

**DOI:** 10.1371/journal.pone.0128716

**Published:** 2015-06-12

**Authors:** Ida Engqvist, Katja Wyss, Charlotte Asker-Hagelberg, Ulf Bergman, Ingegerd Odar-Cederlöf, Carl-Olav Stiller, Jessica Fryckstedt

**Affiliations:** 1 Karolinska Institutet, Department of Medicine, Department of Emergency Medicine, Karolinska University Hospital Solna, SE 171 76, Stockholm, Sweden; 2 Karolinska Institutet, Department of Medicine, Clinical Pharmacology Unit, Karolinska University Hospital, Stockholm, Solna, SE 171 76, Stockholm, Sweden; 3 Medical Products Agency, P.O. box 26, SE 751 03, Uppsala, Sweden; 4 Karolinska Institutet, Division of Clinical Pharmacology, Department of Laboratory Medicine, Karolinska University Hospital, Huddinge, SE 141 86, Stockholm, Sweden; 5 Karolinska Institutet, Centre for Pharmacoepidemiology, Department of Medicine, Karolinska University Hospital Solna, SE 171 76, Stockholm, Sweden; Universidad de Valladolid, SPAIN

## Abstract

**Introduction:**

Correct information on patients’ medication is crucial for diagnosis and treatment in the Emergency Department. The aim of this study was to investigate the concordance between the admission chart and two other records of the patient’s medication.

**Methods:**

This cohort study includes data on 168 patients over 18 years admitted to the Emergency Ward between September 1 and 30, 2008. The record kept by the general practitioner and the patient record of dispensed drugs in the Swedish Prescribed Drug Register were compared to the admission chart record.

**Results:**

Drug record discrepancies of potential clinical significance between the admission chart record and the Swedish Prescribed Drug Register or general practitioner record were present in 79 and 82 percent, respectively. For 63 percent of the studied patients the admission chart record did not include all drugs registered in the Swedish Prescribed Drug Register. For 62 percent the admission chart record did not include all drugs registered in the general practitioner record. In addition, for 32 percent of the patients the admission chart record included drugs not registered in the Swedish Prescribed Drug Register and for 52 percent the admission chart record included drugs not found in the general practitioner record. The most discordant drug classes were cardiovascular and CNS-active drugs. Clinically significant drug record discrepancies were more frequent in older patients with multiple medication and caregivers.

**Conclusion:**

The apparent absence of an accurate record of the patient’s drugs at admission to the Emergency Ward constitutes a potential patient safety hazard. The available sources in Sweden, containing information on the drugs a particular patient is taking, do not seem to be up to date. These results highlight the importance of an accurate list of currently used drugs that follows the patient and can be accessed upon acute admission to the hospital.

## Introduction

Many physicians in the Emergency Department have experienced the difficulty in obtaining a correct medication history. Incomplete knowledge of the patients’ medication may result in incorrect diagnosis and inadequate treatment. In addition, adverse drug reactions, unwanted drug interactions, abrupt discontinuation of medication and other drug related problems (DRPs) may not be identified [1–6]. Physicians can access information about prescribed and dispensed drugs via the Swedish Prescribed Drug Register (SPDR) [7–8]. However, the utility of the SPDR is in an Emergency practice has not been evaluated before. Most patients have a general practitioner (GP), who keeps a medication record (GP record—GPr). Upon admission, the emergency physician registers medications in an admission chart record after interviewing the patient, and/or care-takers without prior consultation of the SPDR or the patients’ GP. Our previous studies have addressed the therapeutic problems generated by insufficient knowledge on the currently used drugs [2–6]. The aim of this study was not to analyse the clinical consequences of drug record discrepancies in our patient cohort, but rather to quantify major discrepancies of potential clinical importance between different sources of information: the admission chart record, the GP record and drugs in the SPDR.

## Methods

The Karolinska University Hospital Solna is one of seven emergency hospitals in Stockholm (2 million inhabitants), Sweden. The Emergency Department has 80 000 visits and 2700 admissions to the Emergency Ward (EW) annually.

Upon admission to the EW, the physician records the patient’s current medication in the electronic patient file [9]. This information is derived from previous hospitalizations, the patient directly, relatives or other caregivers. A commonly used source of information is a medication record acquired from the patient’s GP. Thirdly, as mentioned, via the Swedish Prescribed Drug Register (SPDR) data on all dispensed drugs, but not over-the-counter (OTC) drugs (unless they were prescribed), can now be made available to prescribing physicians after informed consent of the patient [7,8].

### Patients and hospital setting

The study was conducted between September 1 and 30, 2008. Patients considered for inclusion were above 18 years, admitted to the EW during office hours on weekdays. Weekend and night admissions were reviewed on the first subsequent workday if the patients were still present in the ward. Patients signed the consent form after receiving verbal and written information of the study. The following data were recorded: age, sex, medications on admission, cause and length of hospitalization and caregivers outside of the hospital. Patients who declared no medication were also included since we hypothesised that this information may not be correct.

The admission chart record (ACR) was collected from drugs listed in the admission chart (107 patients) or printed from the electronic health record (61 patients) linked to the electronic patient file [9]. Data from the SPDR was acquired for a period of minimum six months prior to admission since most drugs are prescribed on a three or six month basis in Sweden (monthly elsewhere). Patients named their GP, who was informed of the study purpose and requested to provide a record of the patients’ current medication. The GPs were reminded twice. The patients were asked if they consulted several doctors for medications, thus assessing the number of care-givers each patient had.

### Comparison between information on the patients’ medication

Two investigators (IE and KW) collaborated in comparing the generic substances by name in the three records. No comparison was made between the GP record and the SPDR. Dietary supplements, food agents, non-prescription local treatment e.g. tear substitutes and moisturisers, were excluded. A drug that was used according to one record, but was not listed in the other record was classified as a discrepancy.

### Criteria for assessment of the potential clinical importance

A discrepancy between records was judged as of potentially clinical significant by the following criteria:

- The drug was used to treat a condition that needs continuous treatment (for example hypertension).
- The patient could potentially develop a drug related problem when receiving the wrong dosage/regimen of the drug (for example analgesics).
- The patient could develop symptoms on abrupt withdrawal of the drug (for example benzodiazepines or beta-receptor blocking agents).

The drug record discrepancies that did not meet these criteria were analysed by specialists in Internal Medicine and Clinical Pharmacology (JF and CAH).

The drug record discrepancies identified by comparison of the admission chart record and the SPDR were classified according to a system previously described in the pilot study [6].

- A _admission chart-/_ SPDR+ a drug not included in the admission chart record, but dispensed at pharmacy according to the SPDR.
- B _admission chart +/_ SPDR- a drug in the admission chart record but not dispensed at pharmacy according to the SPDR.
- C a drug found in both records with significantly different dose or dosing regimen.
- D a drug found in both records but the prescribed amount according to the SPDR is insufficient to last until the admission date or up to 14 days before admission date.

The drug list discrepancies that could arise in the comparison between the admission chart record and the GP record were classified in a similar manner:

- A _admission chart-/GP+_ a drug not included in the admission chart record, but registered in the GP record.
- B _admission chart+/GP-_ a drug in the admission chart record but not included in the GP record.
- C a drug found in both records with significantly different dose or dosing regimen.

Drugs were grouped according to WHO´s Anatomical Therapeutic Chemical (ATC) classification system [10].

### Statistical Analysis

Graph Pad Prism version 5.02, San Diego, CA was used for statistical analysis. Contingency tables were analysed by Fishers exact test. For pair wise comparison Mann-Whitney U-test was used. Statistical significance was set at p <0.05.

### Ethical considerations

This study was approved by the Regional Ethics Review Board (Regionala Etikprövningsnämnden 2008/3:6) at Karolinska Institutet.

## Results

### Patients

Of the 247 patients admitted to the EW during the study period, 47 were admitted and discharged outside office hours, five declined participation, 25 were unable to give consent and two were foreign citizens. Thus, 168 patients were included. Cardiovascular diseases and dyspnoea accounted for half of the admissions (Table 1a). The median age was 68 years (range 19–93), sex distribution was even (male 83 and female 85), median number of drugs on admission was six (range 0–29) and the median length of hospital stay was three days (range 1–49).

**Table 1:** a. Reasons for admission to the Emergency Ward and b. clinically relevant discrepancies.

a		b.		
Symptoms/diagnosis	No. Patients (%)	Other relevant diagnoses	No. Clinically relevant discrepancies ACR/SDPR (%)	No. Clinically relevant discrepancies ACR/GPr (%)
Bleeding	8 (4.7%)	Cardiovasc 12, HT:19, HF:5, AF:13, DM:9, Ca pulm:2, ethylism:1, haematologic disease:2, ulcerationGI:1, renal failure:1, autoimmune disease:1	27,(5.1%)	34 (6.4%)
Cardiovascular diseases MI, arrhythmias, angina, HF	47 (28%)	COPD:15, Asthma:1, acutebronchitis:1, embolipulm:1, metpulm.5, HF:5,cardiovasc 2, HT:3, AF:3, empyema: 1	133 (25%)	135 (25%)
Diabetes Mellitus	3 (1.8%)	Syncope:11, Ca corpora uteri:1, SLE, nephritis:1, HT:3, Venous ulcer:1,COPD:1, osteoporos:1, anemia 1, pleuritis:1, op heart valve: 1, anxiety:1 depression:1, Alzheimer:1, met pulm:2	13 (2.4%)	2 (0.4%)
Dizziness Vertigo, Syncope, Falls	19 (11%)	Migraine:1, hepatitis C:1, liver cirrhosis 2,: methadone pancreatitis:2, ethylism:1, appendicitis;1, DM:2, HT:2,HF:2, AF:2 angina:1, RA:1, post stroke:1, pneumonia:1, ulcuceration: 2, gastritis:1	50 (9.4%)	53 (9.9%)
Dyspnea	38 (23%)	Cardiovascular dis: 5,Mb Crohn:2, COPD:2,DM:1, liver ca:1, ALS:1, hematologic disease: 1, KLL:1, angina p:1,	152 (28%)	180 (34%)
Impaired general condition	18 (11%)	Renal failure: 2, ulcerative legs: 3, perianal abscess:1, hyper- hypoglycemia:3, hypothermia:1, cough:2, traffic incidence:1, nausea:1	59 (11%)	70 (13%)
Infections	10 (5.9%)	Cancer:2, DM:1, COPD:2, AF:1, HF:2, HT:3, cardiovascular disease:1, vertebral fracture: 1, renal failure:3	25 (4.7%)	11 (2.1%)
Intoxications Unconsciousness	6 (3.6%)	SLE nephritis:1, DM:3,HT:3, Psychiatric diseases:3, TX:1, HD:1	19 (3.6%)	17 (3.2%)
Miscellaneous	6 (3.6%)	Rectal bleeding:3 ulcerative colitis:1, RA:1, GI bleedings:2, haematemesis:1, urinary cancer:1, DM:1, COPD:1	26 (4.9%)	16 (3.0%)
Pain	13 (7.7%)	HT:1, renal failure:1 vascular disease:1, RA:1, HT:1, AF:1	29 (5.4%)	16 (3.0%)
Total	168 (100%)		533 (100%)	534 (100%)

Abbreviations: No. = number of patients with this diagnosis at admission (Table 1a) and number of clinically relevant discrepancies (Table 1b).

COPD = chronic pulmonary disease, HT = hypertension, HF = heart failure, AF = atrial fibrillation, MI = myocardial infarction RA = rheumatoid arthritis, SLE = systemic lupus erythematosus,TX = transplantation, DM = diabetes mellitus, HD = hemodialysis, ALS = amyotrophic lateral sclerosis, ca = cancer, met = metastases, pulm = pulmonary

When comparing the admission chart record and the SPDR, 675 drug record discrepancies were identified, 533 (79%) classified as clinically significant. At least one drug record discrepancy was noted in 141 patients (84%), and 133 patients (79%) had at least one clinically significant drug record discrepancy. The mean number of clinically significant drug record discrepancies per patient were 3.2 (median 2.5, range 0–13) (Table 2).

**Table 2:** Number of discrepancies comparing the records from the admission chart (ACR), the Swedish Prescribed Drug Register (SPDR), and the General Practitioner register (GPr).

No. of drug list discrepancies	ACR / SPDR	ACR / GPr
	No. of patients (%)	No. of patients (%)
0	35 (21%)	21 (18%)
1–3	70 (42%)	29 (24%)
4–6	40 (24%)	40 (34%)
7–9	15 (9%)	13 (11%)
>10	8 (5%) max 13	16 (13%) max 23
sum	168 (100%)	119 (100%)

The most common (54%) clinically significant drug record discrepancy was drugs dispensed according to the SPDR but not found in the admission chart record

(type A _admission chart-/_ SPDR _+_). This concerned 106 patients (63%).

Drugs being registered in the admission chart record but not present in the SPDR

(B admission chart+/SPDR-) constituted 18% of the total number of clinically significant drug record discrepancies, affecting 53 (32%) of the patients.

### Comparison of admission chart record with the GP record

According to 131 patients, a GP prescribed their medications. Twelve GPs did not provide a medication record, leaving 119 patients available for evaluation.

Of the 626 identified drug record discrepancies, 534 (85%) were classified as of potential clinical significance. In 106 patients (89%), at least one discrepancy was noted, of which 82% (98 patients) were judged to be clinically significant rendering an average of four clinically significant drug record discrepancies per patient (median 3, range 0–23)(Table 2).

A drug present in the admission chart record but not in the GP record (Type B admission chart +/GP- discrepancies) was most common and accounted for 50% of the clinically significant drug record discrepancies, affecting 62 (52%) patients. A drug being present in the GP record but not in the admission chart record (Type A admission chart-/GP+,) accounted for 40% of the clinically significant drug record discrepancies, affecting 74 (62%) patients. A significant difference in dose or dose regime (Type C discrepancies) made up 10% of the clinically significant drug record discrepancies, affecting 35 (30%) patients. (Table 3)

**Table 3:** Distribution of different types of discrepancies between records from the admission chart (ACR), the Swedish Prescribed Drug Register (SPDR), and the General Practitioner register (GPr).

Types of discrepancies	ACR / SPDR	ACR / GPr
	% of discrepancies	% of discrepancies
Type A	54%	40%
Type B	18%	50%
Type C	21%	10%
Type D	6%	-

Type A: medication present in SPDR or GPr but not in ACR.

Type B: medication present in ACR but not in SPDR or GPr.

Type C+D: discrepancy regarding dose/regime (C) or amount prescribed insufficient to last until admission (D) in medication present in both records.

### Patients at risk

Patients with at least one clinically significant drug record discrepancy of the admission chart record and the SPDR or the GP record were older (p < 0.05, Mann Whitney test) and medicated with more drugs (p <0.0001, Mann Whitney test) compared to patients without clinically significant drug record discrepancies (Table 4). Cardiovascular symptoms/diseases and dyspnoea were the most common reasons and accounted for half of all admittances at the EW (Table 1b).

**Table 4:** Potential risk factors for drug discrepancies when comparing records from the admission chart (ACR), the Swedish Prescribed Drug Register (SPDR), and the General Practitioner register (GPr).

	ACR / SPDR		ACR / GPr	
	≥ 1 drug list discrepancies (n = 133)	No drug list discrepancies (n = 35)	≥ 1 drug list discrepancies (n = 98)	No drug list discrepancies (n = 21)
Age, years (mean (SD))	68.2 (SD 16.2)	55. (SD 16.3)	68.9 (SD 15.2)	57.0 (SD 21.7)
Number of medications at admission (mean (SD))	8.3 (SD 6.5)	1.7 (SD 3.1)	7.5 (SD 6.4)	1.5 (SD 2.6)
>1 health care provider	34.6%	14.3%	40.8%	14.3%
Sex, male	48.1%	54.3%	47.6%	46.9%

These symptoms were also linked to the highest rate of discrepancies, which generally were of the same order of magnitude in the GPr and the SPDR (Table 1b). Multiple caregivers were more common in patients with clinically significant drug record discrepancies (p = 0.025, Fishers exact test). There was no difference between men and women regarding the presence of drug record discrepancies (p = 0.5714, Fishers exact test). (Table 4)

### Drugs involved in clinically significant drug record discrepancies

In the comparison between admission chart record and the SPDR, CNS-active drugs were most commonly identified (in total 28%, whereof 62% neuroleptics, sedatives and hypnotics (N05) and 31% analgesics (N02)), followed by cardiovascular drugs (in total 22%, of which 28% were diuretics (C03), 20% beta-blocking agents (C07), and 20% renin angiotensin blocking agents (C09)). A similar picture was seen comparing the admission chart and the GP record, where cardiovascular drugs were the largest group (in total 27%, of which 29% belonged to diuretics (C03), 20% beta-blocking agents (C07), and 20% cardiac therapy (C01), e.g. nitroglycerine) followed by CNS-active drugs (in total 20%, 59% neuroleptics, sedatives and hypnotics (N05) and 29% analgesics (N02)) (Table 5).

**Table 5:** Drugs classified by pharmacological groups according to the ATC classification system [10] involved in discrepancies in records from the admission chart (ACR), the Swedish Prescribed Drug Register (SPDR), and the General Practitioner register (GPr) as the numbers and the percentages of discrepancies.

ATC Drug Class	ACR / SPDR	ACR / GPr
	No. of Discrepancies (%)	No. of Discrepancies (%)
A. Alimentary tract and metabolism	76 (14%)	93 (17%)
B. Blood and blood forming organs	41 (7.7%)	50 (9.4%)
C. Cardiovascular system	118 (22%)	144 (27%)
D. Dermatological	5 (0.9%)	3 (0.6%)
G. Genito-urinary tract and sex-hormones	18 (3.4%)	14 (2.6%)
H. Hormones	13 (2.4%)	15 (2.8%)
J. Infectious diseases	9 (1.7%)	7 (1.3%)
L. Tumors and immune system	13 (2.4%)	5 (0.9%)
M. Muscular-skeletal system	18 (3.4%)	24 (4.5%)
N. Nervous System	148 (28%)	106 (20%)
P. Anti-parasitic	1 (0.2%)	0 (0.0%)
R. Respiratory system	66 (12%)	64 (12%)
S. Sensory organs	3 (0.6%)	4(0.7%)
V. Various	4 (0.8%)	5 (0.9%)
Total	533 (100%)	534 (100%)

## Discussion

As part of ongoing studies of drug related problems at the Karolinska University Hospital, Huddinge and Solna [2–6] we have quantified drug record discrepancies of potential clinical importance between different sources of information regarding current medication for patients admitted to the emergency ward.

We found that 79% of the included 168 patients had at least one clinically significant drug list discrepancy when comparing the admission chart record to the SPDR.

A similar picture was seen in the 119 patients for whom the admission chart was compared to the GP record. This indicates that a majority of patients are subject to a continuance gap in their preadmission medication regimen. This corresponds well with the findings of Ekedahl et al [11] who compared the admission chart medication list and the medications reported by the patients, to the SPDR in a similar Swedish population.

In our study, older patients using many drugs prescribed by multiple caregivers were more prone to clinically significant discrepancies. It can be hypothesized that these factors contribute to the comparatively large number of ADRs found in elderly patients [2, 4–6].

Patients admitted because of cardiovascular symptoms/diseases or dyspnoea represented the largest percentage of discrepancies (Table 1b). Correspondingly, among the medications used, cardiovascular and CNS active drugs were the most common ones and may be seen as risk factors. These medications are also among the most commonly prescribed drugs in the general population [12], frequently causing DRPs [2–5] which highlights the importance of these particular discrepancies (Table 5).

Some discrepancies may arise because many of these medications may need titration by the GP by verbal instruction, while others are prescribed for use when needed (e.g. nitroglycerine or hypnotics) and may therefore be “forgotten” by the patient when in the EW.

The SPDR is a unique initiative in Sweden, and its’ usability has not yet been evaluated in an emergency setting. The lack of correct information on the patient’s medication and the consequences of this have, however, previously been reported. Several studies have evaluated the accuracy of the admission chart record compared with more thorough medication reconciliation strategies, such as second interviews [1, 2, 6, 9,13], records from local pharmacies [14], accounts of drug dispensers collected from the patients’ home [14–15] and/or records from primary care physicians [15–18]. In accordance with our findings, these have shown that discrepancies are common, seen in between 54–96% of studied patients. Between 27–59% of these drug record discrepancies were considered as potential medical risks. Our results lie in the upper end of the spectrum, possible reasons as to which will be discussed below.

### Discrepancies between the admission chart record and the SPDR

The SPDR does not register OTC drugs (unless they are prescribed), but this only partly explains the large number of discrepancies between these two records. We found that more than 60% of the patients lacked a clinically important prescription drug in the admission chart record. This highlights an important limitation with the SPDR; if a treatment was stopped, the dose adjusted or the drug replaced by another, the SPDR will no longer be accurate.

The opposite problem, a drug present in the admission chart record, but not in the SPDR, was seen in 32% of the patients. Non-compliance, OTC drugs or sporadic medications could explain this. Another possibility is that the admission chart record was based on a previously recorded medication record erroneously assumed by the admitting physician to be correct.

### Discrepancies between the admission chart record and the GP record

One possible reason for the drug record discrepancies could be the organisation of the health care in Sweden. The GP does not have unique responsibility for patients’ medication. Infrequent consultations, multiple caregivers and poor transmission of information from hospital to GP, could explain the lack of concordance between records. In this study we only contacted the patients’ GP, but not other outpatient caregivers.

Multiple caregivers may be a problem in urban areas since health care often is provided by several GPs, private specialists and hospital based doctors. The GPs in the Stockholm area accounted for only one third of medical consultations and prescriptions issued to the population [19]. The GPs share of total consultations and prescriptions is considerably higher in countries where the GPs have a gatekeeper role such as Denmark and the UK, where GP prescribing accounts for the majority of National Health Service drug prescribing [20–21]. All types of drug list discrepancies could also derive from transcription errors and non compliance.

### Limitations of the study

This study was conducted in a tertiary care university hospital where many patients with multiple diagnoses such as advanced heart disease, cancer, renal and liver failure are treated and this may not be representative for hospitals in general. The patient number is small, but representative of the category of patients who are admitted to the EW in this hospital [2–6].

It was not our aim to determine which of the records that was most accurate. This would have required a more complex reconciliation process involving a second interview with the patient or a close caregiver, if at all possible.

In an attempt to assess the patient selection we compared the demographic characteristics of the excluded patients (n = 27) with the study population. No significant differences in age, average hospitalization-days or the number of medications upon admission were identified (data not shown). This comparison does not include 47 patients who were admitted and discharged outside office hours. We cannot rule out the possibility of selection bias. Patients admitted outside office hours but still present in the ward on the subsequent weekday were, however, included in the study, partly compensating for this.

The SPDR automatically registers prescribed drugs that are dispensed [7]. Data was acquired for a period of minimum six months prior to admission, based on the assumption that most drug prescriptions are renewed every 3–6 months. By doing so we might have missed prescriptions renewed annually, but also avoiding registering old and no longer relevant prescriptions. Acquiring data from a shorter time period from SPDR, we could have missed important prescriptions renewed less frequently.

This study did not determine if the drug discrepancies resulted in negative clinical outcome. The maximal follow up period at the hospital was three days and we did not trace the patients after that. In order to assess the clinical consequences of drug record discrepancies we would need a matched control group of patients with a correct list of drugs. In order to have sufficient statistical power, a study like that would have to be of considerable size.

### Consequences

Clinically significant drug record discrepancies can lead to a number of errors in patient care, both during hospitalization and after discharge. DRPs, such as double prescriptions, may arise or simply remain undetected. The SPDR provides accurate information with regard to prescribed drugs only at the moment of drug dispensation, but is not necessarily correct and complete at the time of admission to the EW. National projects, where the most recent prescriptions and changes in dosage of the individual patient’s medication can be accessed by all caregivers at all times, are on the way and much needed [22]. Drug related morbidity is a global problem and one of the most common reasons for hospitalization, particularly in the elderly [23–25].

The availability of data on dispensed drugs such as in the Swedish Prescribed Drug Register and similarly in all Nordic counties [26] is, however, not yet available in many countries. In an extensive review of Nordic publications we have not found any similar studies outside Sweden to compare our results with [27]. However, we have reasons to believe that this is a global problem, maybe less pronounced in settings where the GP has a gatekeeper function [20–21]. Relying on the SPDR as the only source of drug record may potentially jeopardize patient care. The extent of the problem has not yet been determined.

Every patient should have an accurate list of drugs that can be assessed not only upon admission at the emergency ward but also when consulting other healthcare units. Obtaining this goal should have a high priority within any healthcare system.
